# Titles change the esthetic appreciations of paintings

**DOI:** 10.3389/fnhum.2015.00464

**Published:** 2015-08-25

**Authors:** Gernot Gerger, Helmut Leder

**Affiliations:** Department of Psychological Basic Research, Faculty of Psychology, University of ViennaVienna, Austria

**Keywords:** titles, artworks, fluency, facial emg, esthetics

## Abstract

Esthetic experiences of artworks are influenced by contextualizing information such as titles. However, how titles contribute to positive esthetic experiences is still an open issue. Considering that fluency, as well as effortful elaborate processing, potentially influence esthetic experiences, we tested how three different title types—semantically matching (fluent), semantically non-matching (non-fluent), and an “untitled” condition (control)—affected experiences of abstract, semi-abstract, and representational art. While participants viewed title/artwork combinations we assessed facial electromygraphic (fEMG) recordings over M. corrugator supercilii and M. zygomaticus major muscle to capture subtle changes in emotional and cognitive processing, and asked for subjective liking and interest. Matching titles, but also the more effortful untitled condition, produced higher liking compared to non-fluently processed, non-matching titles especially in abstract art. These results were reflected in fEMG with stronger M. corrugator activations in the non-matching condition followed by the untitled condition. This implies high cognitive effort as well as negative emotions. Only in the matching condition, M. zygomaticus was more strongly activated indicating positive emotions due to fluency. Interest, however, was hardly affected. These results show that high levels of dis-fluency and cognitive effort reduce liking. However, fluency as well as moderate levels of effort contribute to more positive esthetic experiences.

## Introduction

Every year millions of visitors attend art exhibitions expecting to have pleasurable experiences. Such experiences might be determined by the artworks, their style or color, but also by the kind of feelings the paintings evoke, the stories they tell, and the semantics they transport (Chatterjee, 2003; Leder et al., 2004; Cupchik et al., 2009). Often artworks are accompanied by titles that aim to support the viewers’ esthetic experience. For example, Levinson (1985) proposed a title to be an invariably significant component which “helps determine [an artwork’s] character, and is not just an incidental frill devoid of import, or a mere label whose only purpose is to allow us to refer to the work and distinguish it from its fellows” (p. 29). The role of titles on understanding (Russell, 2003; Leder et al., 2006; Swami, 2013), time of perception, and visual exploration (Kapoula et al., 2009; Hristova et al., 2011), or liking (Russell, 2003; Belke et al., 2010; Swami, 2013) has been shown in previous studies. Yet, how titles and artworks interact and produce pleasurable states is still an open question.

One approach, fluency theory (Reber et al., 2004) proposes that positive esthetic experiences are linked to processing ease. A higher ease through semantic match or reduced mental effort leads to positive affect and thus more positive liking. A positive link between fluency and liking was shown by Reber et al. (1998) for simple patterns and objects but also for photographs (Tinio et al., 2011) and art-styles (Leder, 2003). Belke et al. (2010) also reported a positive effect of matching titles with works of art indicated by higher liking ratings, in accordance with this explanation.

On the other hand, works of art are often made for challenging the human mind by providing cognitively interesting topics, violating perceptual processing routines, and presenting ambiguous, abstract or perceptually non-salient content (Dutton, 2009; Pelowski and Akiba, 2011) more in accordance with liking for cognitive effort and dis-fluency. This argument also fits more general perception research that shows that dis-fluent processed stimuli due to novelty, complexity, or ambiguity are often liked (Hekkert et al., 2003; Jakesch and Leder, 2009; Marin and Leder, 2013).

In the empirical art studies, research suggests that perceivers may often not have to come to a definitive conclusion or understanding when evaluating art (Leder et al., 2004). Similarly, Van de Cruys and Wagemans (2011) propose that the rewarding effect from the arts stems from an effortful progress toward classification, understanding, and from a transition of a state of initial ambiguity and uncertainty toward a state of increased predictability and certainty. That is, the effortful progress toward more understanding and assigning meaning is rewarded and linked to positive affect and liking. Thus, certain levels of dis-fluency may remain. In a similar vein Martindale (1984) associates the pleasure gained from looking at an artwork with the amount and diversity of cognitive representations activated by its processing. This is in line with Bartlett’s (1932) concept of “effort after meaning” linking perception and liking of art to an effortful process in the search for meaning. According to these accounts it is not ease of processing but rather active processing effort and elaboration (Armstrong and Detweiler-Bedell, 2008; Muth and Carbon, 2013) which is considered pleasurable in the arts. Hence, title/artwork matching might in fact be less of an important factor in appreciating especially more modern or abstract art (Leder et al., 2006; Belke et al., 2010).

To further add complexity to this topic, both fluency and dis-fluency lines of explanations are combined in the recent pleasure-interest model of Graf and Landwehr (2015). They propose that positive esthetic experiences are governed by two consecutive processes – an automatic stimulus-driven, fluency sensitive process followed by an elaborate controlled process linked to dis-fluency reduction. First, a stimulus-driven automatic process measures the discrepancy of the expected vs. actual fluency experience. If this discrepancy is positive, that is, if more fluency is experienced than expected, then a positive affective feeling and in consequence higher liking results. This is in line with the fluency theory by Reber et al. (2004). However, in cases of a negative discrepancy, when the stimulus is less fluently processed than expected, this leads to increases of negative affect. This can decrease liking especially if processing stops at this stage. Importantly, this early automatic stimulus-driven process can be followed by a later elaborate perceiver-driven process which aims to reduce dis-fluency. The motivation to further process the stimulus is particularly pronounced under high levels of experienced dis-fluency. If dis-fluency reduction is successful this later process can modulate the outcome of the earlier automatic processes and lead to more positive esthetic experiences through an increase in esthetic interest.

In turn, previous studies that systematically varied match of titles or information with artworks have reported mixed findings with regard to the above explanations. Lengger et al. (2007) found evidence that matching information is more fluently processed in relation to a no information condition. Matching descriptions reduced brain activations in the left frontal and parietal lobes. This is consistent with studies reporting reduced cortical activation due to processing facilitation in experts (Solso, 2001). Paintings were also rated more meaningful with matching information. However, liking, as expected by the fluency account (Reber et al., 2004), was hardly affected. Similarly, in Leder et al. (2006) only meaningfulness but not liking was affected when artworks were presented with either matching or untitled conditions. Also in Russell (2003, Experiment 1) and Russell and Milne (1997), matching compared to the untitled condition led to higher meaningfulness ratings but also failed to demonstrate increases in liking when a between subject design was employed.

The failure to obtain higher liking ratings could be due to participants’ low levels of a positive fluency discrepancy (Graf and Landwehr, 2015). In a within subject design where fluency discrepancy becomes more salient as the perceiver can directly compare fluency experiences among trials, Russell (Experiment 2) found increases in liking. The experiments of Millis (2001), interestingly, provide evidence for fluency increases but also elaborations (e.g., through dis-fluency reduction) being important for positive esthetic experiences. In Experiment 1 matching title conditions, that were maximally fluent, led to more understanding and higher esthetic evaluations measured by a combined esthetic judgment of liking, interest, number of generated thoughts, and emotions. Results of the second experiment, however, diverge from only fluency increases being influential for positive esthetic experiences. In this experiment participants evaluated artworks with descriptive, elaborative, or non-matching titles which vary from more fluent to less fluent. Elaborative titles led to the highest esthetic evaluations which were followed by the least fluent non-matching condition. Interestingly, descriptive titles received the lowest esthetic evaluations although fluency of processing should be maximal among them. This data pattern is more in line with elaborate processing and dis-fluency reductions being determining positive esthetic evaluations. It should be noted that Millis used a combined general measure of esthetic evaluations. Thus, it is hard to tell whether and how different factors of the esthetic experience were exactly affected by title manipulations.

### Present Study

To test the question of title impact on art liking and engagement, we compared three title conditions – a maximally fluent matching, a maximally dis-fluent non-matching and an untitled condition. We also coupled this with three classes of artworks – representational, semi-abstract, and abstract—in order to account for the possibility that fluency impact may vary with art type or level of abstraction (Lengger et al., 2007; Belke et al., 2010). According to fluency theory effects of matching titles should be strongest for representational paintings as the title refers to the paintings content in a maximal unambiguous manner. Abstract artworks are highly vague in what they represent and there are more possibilities for interpretation. Thus, titles might not facilitate processing in the same manner. Semi-abstract paintings should lay in between as they contain recognizable although often distorted objects (Belke et al., 2010). However, the ambiguities of abstract artworks could also allow for more interpretation opening more possibilities for dis-fluency reduction (Russell, 2003; Leder et al., 2006).

To assess pleasure or art involvement we also employ two separate measures: liking and interest. According to Graf and Landwehr (2015) liking is directly affected by early automatic fluency processes. However, it can be also affected by later elaborate processes linked to dis-fluency reductions through an increase in esthetic interest. Fluent conditions should lead to higher liking. Conversely, non-fluent conditions may also result in higher liking when dis-fluency can be successfully reduced. This should also be reflected in higher interest ratings. That liking increases with higher fluency was shown by Belke et al. (2010). Matching compared to untitled and non-matching conditions led to the highest liking ratings. However, taking a closer look at the results the matching and untitled condition hardly differed. Rather the non-matching condition was devaluated with regard to the other two. This suggests that not only fluency is liked more but that also that dis-fluency is disliked much in line with the assertion of negative fluency discrepancy in Graf and Landwehr (2015). However, esthetic interest was not measured in Belke et al. (2010).

If esthetic liking is determined by increases in fluency then we expect the matching title compared to the non-matching and untitled conditions to have the highest liking ratings. If, however, esthetic liking is also related to reductions of dis-fluency and elaboration then we expect a different result especially in the untitled condition. This condition is less fluent than the matching condition but it allows for a reduction of dis-fluency by elaboration and generating meaning. Hence, in this case untitled and matching title condition should reveal similar liking ratings. The non-matching condition should always produce the lowest liking, as reduction of dis-fluency is hardly possible.

In regards to interest higher interest should be linked to successful dis-fluency reductions. Thus, the non-matching condition which hardly allows for reductions of dis-fluency should have lowered interest ratings (Graf and Landwehr, 2015; see also Silvia, 2005). The untitled condition, however, should allow for dis-fluency reductions and hence we expect higher interest ratings. Importantly, the matching condition might not show strong effects on interest as the title artwork match is maximally fluent and unambiguous (see Jakesch and Leder, 2009). In such cases only small changes in dis-fluency reduction can be expected. Additionally, the motivation to further process the stimulus in an elaborate manner is low in cases of a positive fluency discrepancy (Graf and Landwehr, 2015). Concerning artistic style we expect that abstract artworks with matched titles may uniquely show high ratings of interest as the title might allow for more dis-fluency reduction in abstract art, which by its nature might be more ambiguous (Leder et al., 2006).

There is also a possibility that the effects on interest are rather small: if participants base their esthetic evaluations mainly on early automatic fluency processes but not on later elaborate processes then interest might even not be affected at all. Such a result could be expected in a sample of art naïve participants. Art naïve participants compared to art experts predominantly base their esthetic evaluations on lower order cognitive and emotional processes rather than on higher order complex thoughts and elaborations (Millis, 2001; Leder et al., 2012, 2014). However, in the present study only art naïve participants were tested to exclude strong and heterogeneous effects of expertise due to pre-existing knowledge (Leder et al., 2012). This could override our fluency manipulations through title-artwork matches.

Another limitation overcome in our study is the restriction to behavioral self-reports. Self-reports reflect a summarized outcome of a complex and dynamic decision process. Moreover, individuals may experience both positive and negative effects from fluent/dis-fluent conditions, but this may not show up in behavioral evidence. Therefore, in addition to behavioral assessments of liking and interest, in our study, we included facial electromyographic measures (fEMG) to capture physiological processes associated with the various conditions. Recording fEMG capitalizes on facial muscle movements being sensitive to changes in emotional and cognitive processes over time (Cacioppo et al., 1986; Winkielman and Cacioppo, 2001; Scherer and Ellgring, 2007; Gerger et al., 2011). Activations of the smiling muscle, the M. zygomaticus major, indicate positive emotional processes and activations of the frowning muscle, the M. corrugator supercilii indicate negative emotional processes (Lang et al., 1993; Dimberg and Thunberg, 1998). Interestingly, the M. corrugator supercilii also reacts with more relaxation to positive emotional processes (Larsen et al., 2003). It has been shown that both muscles are sensitive in indicating liking with M. zygomaticus major being stronger activated for liked stimuli and M. corrugator supercilii being stronger activated for disliked stimuli (Gerger et al., 2011; Principe and Langlois, 2011). Importantly, fEMG activations are also responsive to changes in cognitive processing (Scherer and Ellgring, 2007). For example, higher cognitive load manifests in stronger M. corrugator supercilii activations (Lishner et al., 2008). This procedure also allows for a continuous measure of changes in reaction.

Thus, it is a good counterpoint for behavioral measures providing both a more implicit, dichotomous assessment of positive and negative affect and also allows to capture dynamic aspects of the decision process. Regarding hypotheses for fEMG, the model of Graf and Landwehr (2015) predicts early automatic stimulus related fluency reactions and later elaborative processes linked to dis-fluency reductions. This may also especially involve title manipulations leading to automatic early positive or negative emotional reactions and later on-going attempts to reduce dis-fluency. Therefore, if esthetic liking is determined by increases in fluency then we expect the matching title compared to non-matching and untitled condition to lead to stronger M. zygomaticus major activations (Winkielman and Cacioppo, 2001; Gerger et al., 2011), and a relaxation of the M. corrugator supercilii (Topolinski et al., 2009). As these fluency processes are automatically triggered (Graf and Landwehr, 2015) they should appear early after stimulus onset. Such early effects due to fluency might only be short lasting as found in Winkielman and Cacioppo (2001). They showed significant differences in the M. zygomaticus major only in the second after stimulus onset but not in later time bins. Additionally, if dis-fluency has an effect then we expect M. corrugator supercilii to be more strongly activated particularly, in the non-matching condition which is maximally dis-fluent.

If esthetic liking is also related to reductions of dis-fluency and elaboration then we expect the non-matching condition to show long lasting changes in the M. corrugator supercilii due to ongoing higher cognitive load and negative emotional processes. The untitled condition could also result in higher cognitive load and dis-fluency leading to stronger M. corrugator supercilii activations compared to the matching condition particularly in early stages of processing. If load and dis-fluency can be successfully reduced then M. corrugator supercilii activity should eventually decrease over time.

More generally with regards to time course, if participants base their esthetic evaluations mainly on early automatic fluency processes then we expect to find corresponding changes early in fEMG and particularly in liking ratings. On the other hand effects of elaboration are expected to be seen in later components of fEMG and in interest ratings.

## Materials and Methods

### Participants

Participants were students of psychology from the University of Vienna recruited in different introductory courses. Forty-seven participants took part in the experiment in exchange for course credit. Based on a pre-screening questionnaire (Belke et al., 2010) only participants naïve to art were selected as esthetic experiences strongly vary with pre-existing knowledge and expertise (Leder et al., 2012, 2014). Data of eight participants could not be analyzed due to technical problems during recording (2) or due to amount of artifacts in EMG recordings (6). The final sample consisted of 39 female participants (mean age = 23 years; SD = 5.36; range = 19 to 49 years).

### Stimuli

The stimuli consisted of 63 artworks – 21 abstract, 21 semi-abstract cubist, and 21 representational artworks. All paintings were standardized in size to a height of 950 pixels and displayed on a LCD monitor (Nec MultiSyncLCD 3090 WQXi, 31”) with a monitor resolution of 2400 × 1200 pixels.

Artworks/title combinations were chosen according to pre-studies: our study design afforded either semantically matching or non-matching title/artwork combinations. The semantically matching titles consisted of self-generated titles by the authors which related to the content of the artworks (e.g., in case of the abstract painting Line NR 48 by Zdenek Sykora the title was “colored circles” referring to the depicted circles; for the representative Edward Hopper (1940) painting “Gasoline” depicting a fuel station the matching title was “fuel station”). For the non-matching titles the generated titles were randomly assigned to the paintings so that either a semantic or conceptual fit was prevented (e.g., for the colored circles the non-matching title was “Iron man” and “Run” for the Hopper painting). The titles differed in length in between 3 to 18 characters (median length: 11 characters, for a list of title/artwork combinations, see Supplementary Table S1).

In a pre-study (16 participants; *M* = 23.2 years, SD = 4.53), match for all title/artwork combinations was rated on seven-point Likert scale (1 not matching at all, 7 perfectly matching). The mean ratings for the matching titles were *M* = 6.29 (SD = 0.34) for representational, *M* = 5.76 (SD = 0.39) for semi-abstract and *M* = 5.66 (SD = 0.39) for abstract paintings. For the non-matching titles, mean was 2.37 (SD = 0.51) for representational, *M* = 2.10 (SD = 0.42) for semi-abstract and *M* = 2.19 (SD = 0.46) for abstract paintings. Match significantly differed between matching and non-matching titles, *F*(1,15) = 1391.8, *p* < 0.001; $\eta_{p}^{2}$ = 0.98, but also within art styles *F*(2,30) = 22.62, *p* < 0.001; $\eta_{p}^{2}$ = 0.60, for the interaction of art style × match, *F*(2,30) = 2.28, n.s., $\eta_{p}^{2}$ = 0.13. Representational artworks received the highest and abstract artworks the lowest match ratings. This can be expected, as abstract art leaves open more possibilities for interpretation compared to more representational art.

Additionally, all paintings were rated on valence (1 very negative, 7 very positive) and liking (1 do not like at all, 7 like it very much) in a separate pre-study (102 participants, 64 female, *M* = 26.9, SD = 7.73), originally designed to prepare artworks for various experiments by our team. The present study, included paintings of rather neutral valence. The mean ratings for each style were near the midpoint of the scale - semi-abstract (*M* = 3.64; SD = 0.63), representational (*M* = 4.19; SD = 0.54), abstract (*M* = 4.16; SD = 0.55). Valence among styles significantly differed – *F*(2,202) = 52.1, *p* < 0.01; $\eta_{p}^{2}$ = 0.33. Abstract and representational were evaluated slightly more positive than semi-abstract paintings (all *p’*s < 0.01, pairwise comparisons, Bonferroni adjusted). Liking also differed in between art-styles, *F*(2,202) = 18.6, *p* < 0.01; $\eta_{p}^{2}$ = 0.15, with the following means: representational: *M* = 4.17; SD = 0.88; abstract: *M* = 3.85; SD = 0.99; semi-abstract: *M* = 3.50; SD = 0.97. Representational artworks were liked more than abstract (*p* < 0.05) and semi-abstract ones (*p* < 0.01) and abstract artworks were liked more than semi-abstract ones (*p* < 0.01, pairwise comparisons, Bonferroni adjusted). Liking ratings were around the mid-point of the scale. This is important as it allows for variations in liking due to our title manipulations.

In the main study we employed a within subject designs to be able to capture subtle effects due to title manipulation (Russell, Experiment 2). Thus, title manipulation was varied within participants. Additionally, we wanted to prevent that the same artwork would be presented multiple times but with different titles. This would render the experimental manipulation obvious and also bears the danger of inventing mere-exposure effects (Zajonc, 1968). Thus, the artworks within each art-style were divided in three equal sized groups of seven artworks. Valence did not differ within these groups. Each group was either presented with a matching, non-matching, or an “untitled” title. Title to artwork assignment was counterbalanced across participants.

### Procedure

All testing confirmed with the ethical standards of the University of Vienna. Upon arrival participants were greeted, all experimental procedures were explained, and then they were asked to sign a consent form. To prevent unwanted priming (demand characteristics), participants were left unaware about the purpose of recording facial EMG by telling them that skin conductance responses would be recorded (Dimberg et al., 2000; Gerger et al., 2011). Facial EMG application conformed to the standards of Fridlund and Cacioppo (1986). Electrodes (4 mm diameter, 7 mm housing) filled with electrode gel (Signa Gel, USA) were placed over the M. zygomaticus major and M. corrugator supercilii region in a bipolar manner on the left side of the face. Before attaching the electrodes, skin was cleaned with alcohol and rubbed with abrasive gel to reduce impedances below 10 kΩ. The ground electrode was placed over the right mastoid. After electrode placement participants were comfortably seated approximately one meter in front of the presentation screen (Nec MultiSyncLCD 3090 WQXi, 31”, resolution 2400 × 1200 pixels) and electrodes were connected to the amplifier (TMS International Portilab 20 channel amplifier, www.tmsi.com, Netherlands). All experimental instructions were provided on screen. Additionally, participants were verbally instructed to prevent extensive movements unrelated to experimental purposes during critical phases in the EMG recording block (e.g., chewing, touching the electrodes, blinking, talking to themselves).

An experimental trial started with a fixation cross (2000 ms), followed by the title (2500 ms). The title was presented centrally on the screen in a size of 36 pixels. After the title, the screen was left blank for 2000 ms, so that any immediate effects due to title presentation could wear off (e.g., cognitive orienting, reading), before the painting was shown for 4000 ms. After the painting disappeared participants provided their ratings. First participants rated how much they liked the painting (not at all 1, very much 7) and then on a new screen how interesting they found the painting (not at all 1, very much 7). Before the next trial an inter-stimulus interval of 6000 ms followed. Artworks title combinations were presented in a randomized order to the participants. After the last trial the participants were thanked and fully debriefed about the purpose of the study.

#### Facial EMG Signal Analyses

Facial EMG signal analyses confirmed standard procedures (Fridlund and Cacioppo, 1986; Van Boxtel, 2001). The data were sampled with 2048 Hz, filtered with a 20 Hz high-pass, 500 Hz low-pass, and 50 Hz notch filter to reduce power line artifact, full wave rectified and smoothed with a moving average filter of 125 ms. All data processing and filtering were applied oﬄine using EEGLAB Toolbox (Delorme and Makeig, 2004). Data were then z-transformed within muscles and participants (Fridlund and Cacioppo, 1986). Thus, EMG data represent relative activation changes in the facial muscles due to stimuli and condition. Additionally video of the participants was recorded to enable oﬄine artifact encoding due to movements unrelated to experimental demands (coughing, sneezing, touching the electrodes, chewing, etc…). Trials containing artifacts were excluded from analyses and participants excluded if no artifact free trials remained in one of the title/artwork condition combinations. Data during stimulus presentation (4000 ms) were then averaged over consecutive 500 ms time bins in relation to a one second pre-stimuli baseline during the fixation cross and then analzsed with IBM SPSS statistics 20 package.

### Behavioral Data

The liking and interest data can be seen in **Table 1**. Data represent the averaged ratings (liking, interest) over conditions and participants. Data were then analyzed separately for the liking and interest ratings by calculating repeated measures analyses of variance (RM-Anova) with the factors title (3: matching, untitled, non-matching) and art style (3: abstract, representational, semi-abstract) in a full factorial model. In cases of violations of sphericity, Greenhouse–Geisser corrections were applied. This can be seen in the corrected degrees of freedom. Additionally, we performed RM-Anovas with the factor title (3) separately for each art style.

**Table 1:** Mean ratings and SD of liking and interest ratings, separately for stimulus type and title.

		Matching	No-title	Non-matching	Style overall
Liking	Abstract	4.25^a,h^ (0.97)	4.11^d^ (1.17)	3.86^a,f^ (1.12)	4.07^i^
	Representational	3.99^c^ (0.90)	4.04^e^ (1.14)	3.99^g^ (0.99)	4.01^j^
	Semi-abstract	3.18^h,c^ (0.93)	3.27^d,e^ (0.97)	3.09^f,g^ (0.85)	3.18^i,j^
	Overall	3.81^b^	3.80	3.64^b^	
Interest	Abstract	4.15^h^ (0.95)	3.92 (1.11)	3.92 (1.15)	3.99^i^
	Representational	3.78 (1.05)	3.96^e^ (1.01)	3.96 (1.12)	3.91
	Semi-abstract	3.63^h^ (0.97)	3.59^e^ (1.13)	3.59 (0.93)	3.64^i^
	Overall	3.85	3.87	3.82	

*Cells sharing the same subscript depict differences at a 0.05 level, all pair-wise comparisons (not corrected). Subscript a and b for comparisons of title. Subscripts c to j for comparisons within art-styles.*

#### Liking Ratings

As can be seen in **Table 1** the matching and untitled condition led to the highest liking ratings. The effect of title manipulation seemed to be strongest for the abstract artworks, but weaker for the other two art styles. We found that title manipulation influenced liking in the predicted direction, *F*(2,76) = 3.23, *p* = 0.045, $\eta_{p}^{2}$ = 0.08, for the main effect of title. This main effect was due to matching titles leading to higher liking compared to non-matching titles (*p* = 0.047, not adjusted) and also a significant trend toward of the untitled condition leading to higher liking than the non-matching condition (*p* = 0.053, pair-wise comparison of factor title, not adjusted).

Additionally, liking differed with art-style, *F*(2,76) = 20.29, *p* < 0.001, $\eta_{p}^{2}$ = 0.35. Abstract and representational artworks were liked more than semi-abstract artworks (pairwise comparisons: *p*s < 0.001, not adjusted). The interaction of art style × title, *F*(4,152) = 1.18, n.s., $\eta_{p}^{2}$ = 0.03, was not significant.

However, as we expected differences between art styles and title manipulation, we additionally analzsed the effect of title within each art style. These analyses yielded a significant effect for abstract artworks, *F*(2,37) = 3.68, *p* = 0.035, $\eta_{p}^{2}$ = 0.16, but not for representational, *F*(2,37) = 0.12, *p =* 0.88, $\eta_{p}^{2}$ = 0.007, or semi-abstract artworks *F*(2,37) = 1.07, *p =* 0.12, $\eta_{p}^{2}$ = 0.05. **Table 1** reports all pairwise comparisons within each factor combination. Taken together, matching an untitled condition led to highest liking with strongest effects found in abstract art.

#### Interest Ratings

As can be seen in **Table 1** and supported by statistical analyses, interest ratings were not significantly affected by title manipulation: *F*(2,76) = 0.91, n.s., *p* = 0.82, $\eta_{p}^{2}$ = 0.01, for the main effect of title. There was only a trend that interest ratings differed due to style, *F*(2,76) = 2.87, *p* = 0.06, $\eta_{p}^{2}$ = 0.07 with abstract and representational artworks receiving higher interest ratings than semi-abstract paintings. The interaction of style × title was not significant, *F*(4,152) = 1.46, n.s., $\eta_{p}^{2}$ = 0.04.

### EMG Data

**FIGURE 1:**
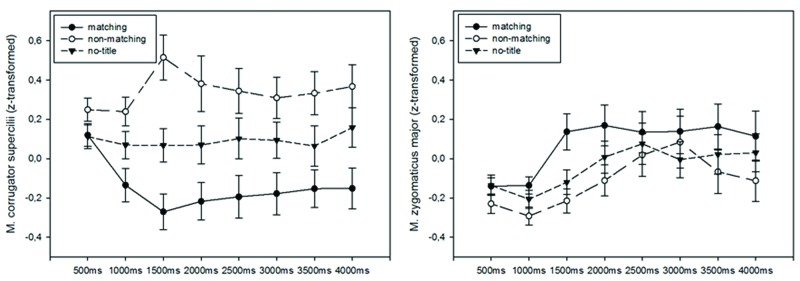
**Activation changes for each title condition averaged over 500 ms time bins for M. corrugator supercilii **(left)** and M. zygomaticus major **(right)**.** Error bars reflect 1 SE of the mean.

The averaged EMG data sampled over participants and conditions were submitted to RM-Anovas, with the factors title (3) × art style (3) × presentation duration (8: EMG activation of eight consecutive 500 ms time bins), conducted separately for the M. corrugator supercilii and M. zygomaticus major. Moreover, to uncover specific changes over time each time bin was analzsed separately. Mean activations of the main factor of title manipulation over time can be seen in **Figure 1**.

#### M. Corrugator Supercilii

As can be seen in **Figure 1** the non-matching condition led to a higher M. corrugator supercilii activation while the matching title condition led to more relaxation as revealed by negative values. The untitled condition was lying in between the other two. This activation pattern was supported by a significant main effect of title, *F*(2,76) = 11.54, *p* < 0.001, $\eta_{p}^{2}$ = 0.23 that was qualified by a title × time interaction, *F*(6.65,252.85) = 2.92, *p* < 0.01, $\eta_{p}^{2}$ = 0.07. To uncover this time course we performed analyses separately for the consecutive 500 ms time bins. Non-matching compared to matching titles led to higher activations from the second time bin to the last time bin (all *p*s < 0.01, Bonferroni adjusted). Non-matching condition differed also from the untitled condition in the third time bin (*p* = 0.007). Additionally, the untitled condition compared to matching condition led to relatively stronger M. corrugator supercilii activations in the second and third time bin (*p* = 0.044 and *p* = 0.006, Bonferroni adjusted).

There were also significant differences due to art style, *F*(2,76) = 11.03, *p* < 0.001, $\eta_{p}^{2}$ = 0.23, and a significant style × time interaction, *F*(7.93,301.26) = 3.49, *p* < 0.001, $\eta_{p}^{2}$ = 0.08 (see **Supplementary Figure S1**), but all of these effects did not interact with title manipulation: style × title, *F*(3.53,9.24) = 2.03, *p* = 0.11, $\eta_{p}^{2}$ = 0.05, style × title × time, *F*(12.48,474.4) = 1.46, *p* = 0.13, $\eta_{p}^{2}$ = 0.04. The main effect of time was non-significant, *F*(3.14,119.4) = 0.53, *p* = 0.67, $\eta_{p}^{2}$ = 0.01. In sum, non-matching titles led to significantly stronger M. corrugator supercilii activations than the other two conditions.

#### M. Zygomaticus Major

**Figure 1** depicts the effect of title on M. zygomaticus activation which had only minor effect on the overall activation pattern. There were neither a main effect of title, *F*(2,76) = 2.11, n.s., *p* = 0.13, $\eta_{p}^{2}$ = 0.05, nor an interaction of title × time, *F*(5.86,9.24) = 1.32, n.s., *p* = 0.25, $\eta_{p}^{2}$ = 0.03, artistic style × title, *F*(3.11,118.25) = 1.51, n.s., *p* = 0.21, $\eta_{p}^{2}$ = 0.05, or artistic style × title × time, *F*(9.51,361.44) = 1.02, *p* = 0.44, $\eta_{p}^{2}$ = 0.026. Nonetheless, separate post-analyses for each time bin yielded a significant difference for matching compared to non-matching titles in the third time bin (*p* = 0.043, Bonferroni adjusted).

Additionally, M. zygomaticus major activation differed with regard to art-style, *F*(2,76) = 17.32, *p* < 0.001, $\eta_{p}^{2}$ = 0.31, time, *F*(2.14,81.13) = 5.96, *p* < 0.01, $\eta_{p}^{2}$ = 0.14, and artistic style × time, *F*(5.75,218.38) = 5.04, *p* < 0.001, $\eta_{p}^{2}$ = 0.12, reflecting bigger differences in later time bins (see **Supplementary Figure S1**). These results show that title manipulations hardly affected activation pattern of the M. zygomaticus major. Only in an early time interval (1000–1500 ms) did matching titles lead to stronger activations.

## Discussion

This study compared how matching titles, non-matching titles, and a no-title condition affect the appreciation of art. The matching and untitled condition led to similar high liking ratings but to reduced liking in the non-matching condition with strongest effects for abstract art. Interest ratings, however, were hardly affected by our title manipulations. Facial EMG activations also showed specific changes with regard to title manipulations. The non-matching condition led to the strongest M. corrugator activations which started early after stimulus onset and could be seen throughout the whole stimulus period (second till last time bin). This long-lasting activation change implies higher negative emotions and higher cognitive load presumably due to attempts in reducing dis-fluency (Graf and Landwehr, 2015). Concurrently, the matching condition led to relaxation of the M. corrugator supercilii and to more activation of the M. zygomaticus major – both effects appeared early after stimulus onset. Additionally, the effect in the M. zygomaticus major was short lasting (only third time bin) and relatively weak. Taken together, these effects imply more positive emotions in the matching condition especially during early time intervals. The values of the untitled condition were in between the other two conditions, with no-titles leading to weaker M. corrugator supercilii activations compared to the non-matching condition but to stronger activations compared to the matching condition. This effect also appeared rather early in the second and third time bin. In line with assumptions from several esthetic theories, this pattern of results emphasizes the interplay of fluency and effortful processing for esthetic evaluations (Chatterjee, 2003; Leder and Nadal, 2014; Graf and Landwehr, 2015).

We assumed that the matching condition should be maximally fluent with regard to the other two conditions. According to Graf and Landwehr (2015) and to Reber et al. (2004) such higher fluency should show up in more positive emotions and higher liking. Our liking and fEMG data support these assumptions. Manipulating fluency with non-art materials, these studies have reported similar results in terms of the direction and time course of the facial activation patterns (Winkielman and Cacioppo, 2001; Topolinski et al., 2009; Gerger et al., 2011). Interesting to note is the high similarity with Winkielman and Cacioppo (2001) and Gerger et al. (2011) in the time course of the M. zygomaticus activation. Although fluency was manipulated quite differently in these studies, through either presentation duration or perceptual priming, they reported significant differences in the same time window as found in our study. Moreover, as in Winkielman and Cacioppo (2001), the effect of an enhanced M. zygomaticus major activation was only short-lived. Regarding the M. corrugator supercilii, differences in activation between matching and non-matching titles showed up even earlier, after 500 ms. This early effect was characterized by more relaxation in the matching condition. These data suggest that fluency through semantically matching titles led to more positive emotions that emerged relatively early after stimulus onset. Such an early onset is in line with Graf and Landwehr’s (2015) assumption of automatic fluency reactions due to positive fluency discrepancy.

However, our data also suggest that not only fluency increases, but also that dis-fluency and higher elaboration influenced esthetic evaluations. If only fluency increases would have played a role, we would have expected to find the highest M. zygomaticus activations and highest liking ratings in the maximally fluent matching condition. This was not the case. Although we found a weak and short lasting effect of increased M. zygomaticus activity in the third time bin only for the matching condition, the matching and untitled condition were rated very similarly for liking. Both were rated higher compared to the non-matching condition with effects being stronger for the matching condition. Thus, artworks in the dis-fluent non-matching condition were rather devaluated with regard to the other two conditions. This effect was reflected in the fEMG activations. The non-matching condition led to the strongest M. corrugator supercilii activations. The early onset of this effect in the second time bin suggests that automatic (dis-)fluency processes are indeed important for the esthetic judgment (Graf and Landwehr, 2015). Additionally, this effect also was stable over time until the end of the analyses period. This long lasting effect could reflect attempts to find meaning and reduce dis-fluency in the non-matching condition (Scherer and Ellgring, 2007; Graf and Landwehr, 2015). The higher M. corrugator supercilii activation in this condition may indicate two different processes affecting the esthetic judgement. It can reflect negative emotional processes due to dis-fluency (Graf and Landwehr, 2015) and an obstruction in finding meaning (Belke et al., 2010). It can also reflect higher cognitive load due to the attempt to reduce dis-fluency (Graf and Landwehr, 2015). Theoretically, higher M. corrugator supercilii activations through cognitive load could have indirectly affected the artworks’ esthetic evaluations as suggested by the facial feedback hypotheses – e.g., non-emotional facial muscle activation of the M. corrugator supercilii results in more negative emotions (Strack et al., 1988; Neal and Chartrand, 2011). Taken together, these processes may have contributed to the liking devaluations observed in the non-matching condition.

The finding that the untitled condition also led to relatively stronger M. corrugator supercilii activations compared to the matching condition in the second and third time bin suggests that this condition was less fluently processed. Nonetheless, the matching and untitled conditions were rated similarly for liking. This demonstrates that higher liking does not only depend on early automatic fluency processes (Leder et al., 2004; Graf and Landwehr, 2015).

Additionally, if fluency increases alone had contributed to liking we would have expected to find the strongest effects for the non-ambiguous representational artworks for which title/artwork match was highest. The effect was, however, strongest for abstract artworks (see also, Leder et al., 2006). The stronger effect for abstract artworks is in contrast to Belke et al.’s (2010) study, which used a similar experimental manipulation. They found the strongest effects for representational artworks and the weakest effects for abstract artworks (see also Russell and Milne, 1997 for similar results). One explanation of why abstract art was liked more with matching titles in our study could be that the titles led to higher understanding (Swami, 2013) and to higher reductions in dis-fluency (Graf and Landwehr, 2015) as compared to the representational artworks. In the representational artworks the matching titles directly describe what is depicted. This might have contributed little to an increase in understanding and dis-fluency reduction. This idea follows Millis (2001) who argues that in order to positively influence esthetic evaluations information has to contribute something extra beyond what can already be inferred from an artwork. Swami (2013) also contends that extra information has to add understanding to increase liking. In the abstract artworks the title also directly referred to the paintings content but the more heterogeneous and ambiguous nature of abstract art might have allowed for increases in understanding (Swami, 2013) and greater dis-fluency reduction (Graf and Landwehr, 2015).

Such greater dis-fluency reduction should reflect in higher interest ratings. Conversely, if reduction of dis-fluency is hardly possible in the non-matching condition then interest should be reduced. However, interest ratings were not affected by the title/artwork combinations. One possible explanation why we did not find variations in interest could be that participants did not fully engage in more controlled perceiver-driven processes as suggested by Graf and Landwehr (2015). Rather their evaluations might have been based on automatic fluency processes. This is in accordance with the early onset of the effects in facial EMG. However, we also found an ongoing transient change in the M. corrugator supercilii for the non-matching titles, which suggests that the artworks were continuously appraised further. Nonetheless, these appraisals might not have been used for the interest judgments, especially as our sample consisted of art naïve participants who hardly rely esthetic evaluations on higher order cognitive thoughts (Leder et al., 2012, 2014). For example, Millis (2001) has shown that matching information more strongly affected art expert’s evaluations compared to laypersons, presumably because art experts evaluate art more deeply and elaborately. Thus our participants might mainly have used the emotional reactions from automatic fluency processes for their judgment. Nonetheless, testing art experts’ interplay of fluent and dis-fluent processing components in their esthetic appreciation remains an interesting challenge for the future.

Alternatively, interest ratings might not have been affected because they were always collected right after the liking ratings. Thus, participants might have used their subjective fluency experience for the liking ratings only but not for the interest ratings. That is, feelings of fluency might have been discounted by the previous liking rating and hence, did not affect interest (Bornstein and D’Agostino, 1994; Unkelbach and Greifeneder, 2013). However, in contrast to this explanation of fluency being discounted in sequential judgments Forster et al. (2015) have shown that fluency manipulation evenly affected two consecutive evaluative judgments. Thus, it has to be tested in future studies whether rating position had an effect on the interest ratings.

Finally, one further aspect of our study relevant for the effects on interest may be linked to the type of titles we employed. Millis (2001) and Swami (2013) reported that esthetic experiences are more strongly affected if they allowed for a deeper elaboration and understanding. Presumably, this would lead to greater dis-fluency reduction. In our study descriptive titles were used, which provided only little elaborate information. We did this in order to maximize the differences in fluency between unambiguously matching and non-matching titles. This was clearly reflected in the strong differences in amount of rated match between the matching and non-matching condition. However, elaborate titles open the possibility for more diverse interpretations and higher ambiguity in title artwork match and thus, greater inter-personal differences. A much higher interpersonal variability would have afforded a different study design using more artwork titles combinations in order to detect meaningful effects in fEMG. Such a much longer experiment comes at the cost of boredom and fatigue obscuring possible effects due to title manipulation. However, it could be expected that elaborate titles particularly influence later processing stages related to higher cognitive thought and understanding (Leder and Nadal, 2014; Graf and Landwehr, 2015). Such higher order cognitive thoughts could affect the time course of fEMG especially in later time areas as shown by Lanctot and Hess (2007). This remains to be tested in future studies.

In sum, this research shows that fluency increases through matching information alone does not provide a conclusive explanatory frame of why art is liked more. Certain levels of dis-fluency may remain, and increases in cognitive effort can be experienced as positively in the arts. So if you want to have your art liked more make sure to produce an interesting tension including definitely some facilitation of the artwork.

## Conflict of Interest Statement

The authors declare that the research was conducted in the absence of any commercial or financial relationships that could be construed as a potential conflict of interest.

## Supplementary Material

Figure S1 **Activation changes for the different art-styles averaged over 500 ms time bins for M. corrugator supercilii (left) and M. zygomaticus major (right).** Error bars reflect 1 SE of the mean.
