# Supplementary material for: Titles change the esthetic appreciations of paintings
Source: Front Hum Neurosci. 2015 Aug 25;9:464. doi: 10.3389/fnhum.2015.00464 (PMC4548445; doi:10.3389/fnhum.2015.00464)
Supplement: Supplementary file 1 [file Table_S1.DOC]

| Style  Appendix 2: List of titles | Artist | Year | Original title (not used) | Matching title | Non-matching title |
| --- | --- | --- | --- | --- | --- |
| representational | Edward Hopper | 1940 | Benzin | Tankstelle | Run |
| representational | Julius Herburger | 1959 | Landungssteg im Regen | Ans andere Ufer | Bunte Kreise |
| representational | Hermann Teuber | 1962 | Eulenspeicher II | Eulenzimmer | Algen im See |
| representational | Wolfgang Mattheuer | 1966 | Elbfahrt | Donauschifffahrt | Die Steppe |
| representational | Steven Izenour | 1979 | Untitled Arizona | Shopping | Center Urwald |
| representational | Anna Musi | 1999 | Gimme a smooch! | Gib mir einen Kuss | Höhlenmalerei |
| representational | Edward Hopper | 1927 | Leuchtturm in Two Lights | Leuchtturm | Klarinettenspieler |
| representational | Edward Hopper | 1927 | Zwei im Gang | Freie Platzwahl | Landweg nach Indien |
| representational | Frits van der Berghe | 1923 | Sonntag | Drei Priester | Modernes Stadtbild |
| representational | Grant Wood | 1930 | Stone City, Iowa | hügelige Landschaft | Riesenrad |
| representational | Arkadij Petrov | 1980 | Liebe | Im Park | Bunte Stadt |
| representational | Richard Lindner | 1966 | Telefon | Geheimdienst | Die Kreuzigung |
| representational | Paul Kirnig | 1934,45 | Bergwerk | Ein Tag beim Bergwerk | Schwarz auf Weiß |
| representational | Per Kirkeby | 1937 | Djupvand | Haus am See | Dickicht |
| representational | Edvard Munch | 1913 | Straße in Kragerö | Sonntag | Der Knall |
| representational | Ludwig Jungnickels |  | Strandleben an der Adria | Am Meer | Karneval |
| representational | Edward Hopper | 1927 | Automat | Einsame Frau | Drachenbrut |
| representational | Edward Hopper | 1928 | Prospect Street, Gloucester | Hauptstraße | Orangetöne |
| representational | Edward Hopper | 1958 | Sonne in einem Café | Im Café am Eck | Skizze |
| representational | Emil Schumacher | 1950 | Küchenherd | Am Herd | fließendes Blau |
| representational | Alexandre Hogue | 1939 | Das gekreuzigte Land | Zur Erntezeit | Sternschnuppen |
| cubistic | Fernand Léger | 1910 | Figure nude in un bosco | Kriegstreiben | Sanfte Hügel |
| cubistic | Fernand Léger | 1916 | Il soldato con la pipa | Eisener Mann | Ans andere Ufer |
| cubistic | George Braque | 1929 | Natura Morte. Le Jour | Frühstückstisch | Abwasser |
| cubistic | Henri Le Fauconnier | 1912 | Paesaggio di Meulan Hardricourt | Landschaft | Donauschifffahrt |
| cubistic | Juan Gris | 1914 | Natura morta con fruttiera e bottiglia d'acqua | Stillleben | Arizona Shopping Center |
| cubistic | Louis Marcoussis | 1912 | Natura morta con scacchiera | Spiele | Gib mir einen Kuss |
| cubistic | Pablo Picasso | 1919 | Zwei Harlekins | Freundschaft | Fließender Himmel |
| cubistic | George Braque | 1917 | Frau mit Mandoline | Frau mit Instrument | Freie Platzwahl |
| cubistic | Jean Metzinger | 1913 | Donna con ventaglio | Frau mit Fächer | Konservendose |
| cubistic | Juan Gris | 1913 | Le tre carte | Die drei Karten | hügelige Landschaft |
| cubistic | Pablo Picasso | 1917 | Die Italienerin | Die Italienerin | Im Park |
| cubistic | Pablo Picasso | 1909 | Fabbrica a Horta de Ebro | Fabrik unter Palmen | Geheimdienst |
| cubistic | Pablo Picasso | 1909 | Frau in Grün | nachdenkliche Frau | Tanzende Schar |
| cubistic | Pablo Picasso | 1908 | Lady with a fan | Flamencotänzerin | Haus am See |
| cubistic | Auguste Herbin | 1911 | Veduta di un villaggio in collina | Die Dorfkirche | Auf hoher See |
| cubistic | Jean Metzinger |  | Donna con chitarra | Frau mit Gitarre | Am Meer |
| cubistic | Pablo Picasso | 1921 | Die drei Musikanten | Die Straßenmusiker | Einsame Frau |
| cubistic | Pablo Picasso | 1909-10 | Ritratto di Ambroise Vollard | Denkender Mann | Hauptstraße |
| cubistic | Pablo Picasso | 1913 | Stillleben mit Fischen am Fenster | Fische | Sonnenkleid |
| cubistic | Roger de La Fresnaye | 1911 | Officina a La Ferté-sous-Jouarre | Fabrik vor der Stadt | Am Herd |
| cubistic | Juan Gris | 1912 | Omaggio a Paoblo Picasso | Der Maler | Zur Erntezeit |
| abstract | Sol LeWitt | 1960 | Run | Run | Stille |
| abstract | Zdenek Sykora | 1987 | Linien Nr 48 | Bunte Kreise | Eisener Mann |
| abstract | De Es Schwertberger | 2005 | Dickicht | Algen im See | Frühstückstisch |
| abstract | De Es Schwertberger | 2006 | Spielraum 3 | Bubbles | Linien |
| abstract | Emil Schumacher, | 1971 | B-30 | Kontur auf Gelb | Globus |
| abstract | Emil Schumacher | 1958 | Gingo Höhlenmalerei | Grünes | Quadrat |
| abstract | Endre Rozsda | 1942 | Souvenir de la marine | Klarinettenspieler | Kette |
| abstract | Beatriz Milhazes | 2008 | Sinfonia Nordestina | liebliche Symphonie | Wut |
| abstract | Takashi Murakami | 2001 | Tan Tan Bo | Bunter Blick | Hagelsturm |
| abstract | De Es Schwertberger | 1995 | Begeisterung | Lichtblicke | Die drei Karten |
| abstract | Emil Schumacher | 1954 | Steigen und Fallen | Bunte Stadt | Ruhige See |
| abstract | Endre Rozsda | 1974 | Symbole hermétique | Die Kreuzigung | Fabrik unter Palmen |
| abstract | Franz Kline | 1955 | Wanamaker Block | Schwarz auf Weiß | Spirale |
| abstract | Jackson Pollock | 1948 | Dipinto | Dickicht | Das Tor |
| abstract | Endre Rozsda | 1980 | Solitude | Der Knall | Die Dorfkirche |
| abstract | Jackson Pollock | 1947 | Guerra | Haufen | Blau |
| abstract | Jackson Pollock | 1943 | La Donna lunare spezza il cerchio | Drachenbrut | Erdloch |
| abstract | Mark Rothko | 1968 | Ohne Titel | Orangetöne | Grau |
| abstract | Renate Lohrmann | 2004 | Hummingbird | Skizze | Fische |
| abstract | Thomas Reinhold | 1997 | Ortung | fließendes Blau | Goldkästchen |
| abstract | Jill Mason | 2008 | The Road | Ab nach Oben | Der Maler |
