# Supplementary figures and images for: Titles change the esthetic appreciations of paintings
Source: Front Hum Neurosci. 2015 Aug 25;9:464. doi: 10.3389/fnhum.2015.00464 (PMC4548445; doi:10.3389/fnhum.2015.00464)

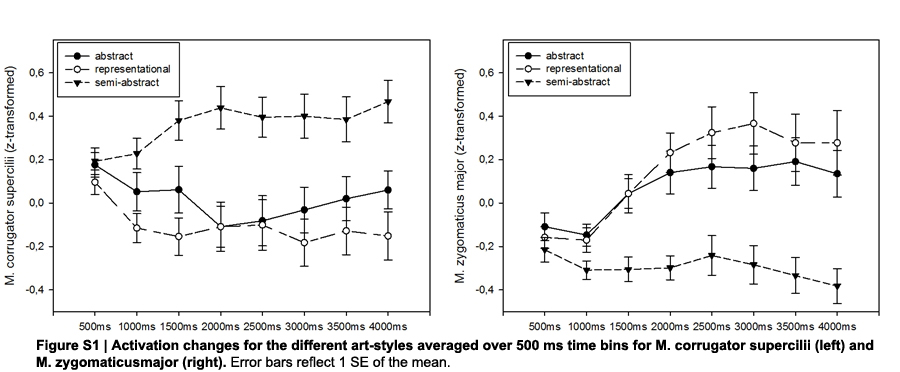

Supplement: Figure S1 — Activation changes for the different art-styles averaged over 500 ms time bins for M. corrugator supercilii (left) and M. zygomaticus major (right). Error bars reflect 1 SE of the mean. [file Figure_S1.JPEG]
